# The effect of loading time on marginal bone change of implants immediately placed after extraction: a retrospective study

**DOI:** 10.1186/s40729-022-00442-2

**Published:** 2022-10-04

**Authors:** Sung-Jae Lee, Euy-Hyun Kim, Dong-Keon Lee, In-Seok Song, Sang-Ho Jun

**Affiliations:** Department of Oral and Maxillofacial Surgery, Korea University Anam Hospital, 73, Goryeodae-Ro, Seongbuk-Gu, Seoul, Republic of Korea

**Keywords:** Dental implant, Immediate implantation, Loading protocol, Immediate loading, Marginal bone change, Biological width

## Abstract

**Background:**

The purpose of this study is to compare and analyze the treatment outcomes between two groups which are both immediately placed implant cases, one is immediate loading, and the other is conventional loading group.

**Methods:**

Medical records of the patients who underwent implant treatment which were immediately placed after tooth extraction were analyzed. Demographic data were collected and by using periapical or panoramic radiographic image, marginal bone level and distant crestal bone level were measured. Marginal bone change over time was analyzed and compared between immediate loading group and conventional loading group.

**Results:**

A total of 71 patients, 112 immediately placed implants after tooth extraction were initially involved. Measuring was done with implants which had not failed (81). 10 implants were had failed and removed. The others were excluded because of follow-up loss, absence of radiographic image, etc. Demographic data were collected, and measured values were averaged at each follow-up and showed in linear graphs.

**Conclusions:**

In case of immediate implantation of dental implant after extraction, loading time could affect marginal bone level or biological width of the implant. Immediate loading group showed 0.92 mm (mean value) more bone loss compared to conventional loading group at bone–implant contact points 24 months after implantation. At distant crestal points, there was no noticeable difference in bone change pattern between two groups.

## Background

Along with conventional prosthetic restorations, dental implants have been chosen for reliable treatment option for partial or full edentulism. When a tooth is lost for any reason—due to periodontitis, deep caries cavity, traumatic event, etc., the alveolar bone volume soon changes continuously after the extraction. Carlsson et al. observed 23% alveolar volume loss at the first 6 months after tooth extraction and followed by additional 11% volume loss after 5 years [1–3]. Lekovic and Schropp also reported that alveolar bone usually shows 2 mm vertical and 6 mm horizontal resorption within 6 months after the extraction [4, 5].

Loss of alveolar bone volume, both vertical and horizontal aspects, are inevitably increases difficulty of placing implant into an appropriate position, which can lead to poor esthetics of an implant prosthesis or can cause undesirable lateral force to an implant fixture. In this regard, Barzilay et al. reported a pilot study which suggested successful osseointegration can be achieved by immediately placing dental implant into extraction socket of long-tailed macaque before significant alveolar bone loss occurs [6]. After the pilot study, there were numerous studies and trials which showed strong evidence of successful prognosis of immediately placed implant into a fresh extraction socket [7]. However, even though the original idea and its purpose of immediately placed implant was to prevent volume loss of alveolar bone after extraction, complete prevention of bone loss is still regarded as hard to be achieved [8–11].

The concept of immediately placed implant is not only to minimize alveolar bone loss after tooth extraction, but also to shorten the total rehabilitation period. In this regard, investigators and clinicians have been introduced modified loading protocols in addition to immediate placement of the implant. Regarding the loading protocol, numerous studies have reported over the past 20 years that there were minimal or no difference between the result of trans-mucosal implants and submerged implants and therefore eventually the immediate loading protocol was introduced [12–14]. Several critical conditions should be satisfied prior to immediately provisionalize the immediately placed implant. The ideal primary stability or initial(insertion) torque value is over 20 to 30 Ncm, and for the protection of fixture from micro-movement which can lead to failure of osseointegration especially at the period of so-called ‘stability dip’, which is the weakest period after the implant placement and before becoming rigid after secondary stability with new bone formation is obtained, occlusal clearance should be given at least 50 to 100 µm, and any parafunctional factors should also be removed while this provisionalization procedure [15].

There were only few studies regarding the treatment outcome according to different loading protocols in patients who have immediately placed implants after extraction. The purpose of this study is to compare and analyze the treatment outcomes between two groups which are both immediately placed implant cases, one is immediate loading group and the other is conventional loading group. Conventional loading in this study, was defined as the cases which implant prostheses were made and restored over 12 weeks after its fixture placement.

In this retrospective study, we evaluated and compared the prognosis of implants according to two different loading protocols by the means of measuring marginal bone level (MBL). All implants were immediately placed after extractions, if with mobility, pain, infection, severe peri-implant radiolucency after its placement regarded as failed implants.

## Materials and methods

Medical records of the patients who visited department of oral and maxillofacial surgery, Anam hospital, Korea university Medicine, from January 2010 to December 2019, and underwent implant treatment which were immediately placed after tooth extraction were analyzed. Among them, only the internal connection type was selected for the implant prosthesis. Following information were collected—gender, age, implanted site, whether the bone grafting was done simultaneously, whether the patients had underlying systemic disease, period from implantation to functioning (loading) as weeks. Height and diameter of implanted fixture was recorded. Age of the patients was investigated, and were divided into two subgroups as elderly patients (more than 65) and below 65. Implanted sites were divided into four groups as maxillary anterior, maxillary posterior, mandibular anterior, mandibular posterior. Failed implants were removed and excluded from the analysis regarding marginal bone level (MBL), their data were separately organized and analyzed.

The collected data were again sorted as following criteria: (1) patients not having severe, or uncontrolled systemic disease (ASA class 1 or 2); (2) patients not having metabolic bone disease such as osteoporosis or not under administration of bone metabolic medications; (3) non-smokers (ex-smokers were also excluded); (4) internal connection type implants. Exclusion criteria were as following: (1) implants which were not properly positioned, overly deep or shallow compared to adjacent alveolar bone level on radiographic image (first, coronal thread is over 2 mm deeper than adjacent alveolar crest level, or exposed over alveolar crest level); (2) incomplete cases which were not finally restored with definite prosthesis; (3) poor radiographic image quality due to inadequate radiographic exposure setting or insufficient radiopacity of graft materials, etc.

Size of implant hardware was grouped by their diameter and height. Implants shorter than 8 mm were grouped as short implant, from 8 to 11.5 mm as standard, longer than 11.5 mm were grouped as long implant. Implants with a diameter of 4 mm or less were grouped as mini, of more than 4 mm and less than 5 mm as standard, of 5 mm or more were grouped as large implants.

**Fig. 1:**
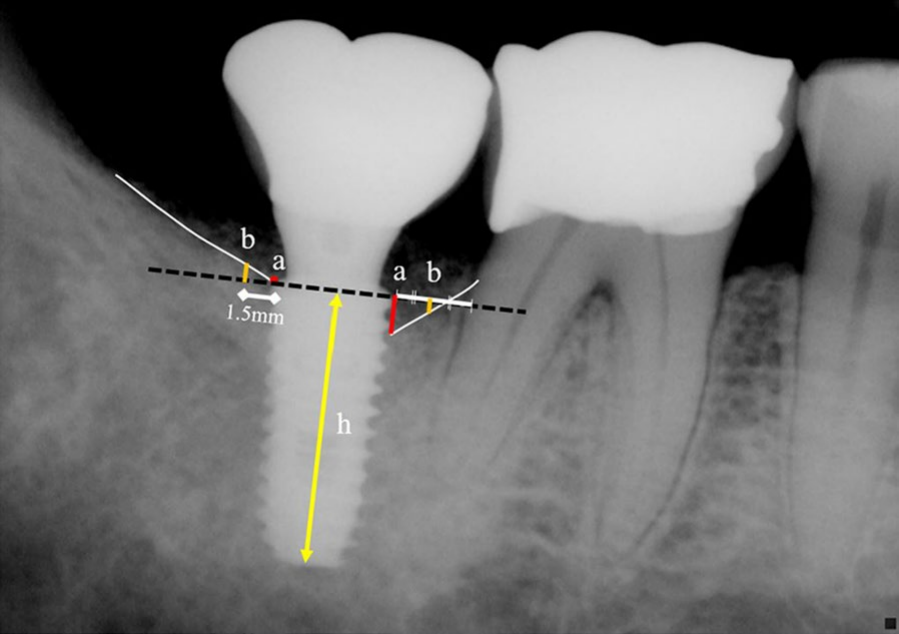
On periapical radiographic image, fixture length (*h*) and marginal bone levels at direct bone–implant contact points (*a*) and distant crestal points (*b*) were measured using image processing program (Image J, National Institute of Health). Using measured lengths, actual marginal bone level was calculated by proportional formula [*H*:*X* = *h*:*a*(or *b*)] (*H* = known actual length of implant fixture, *X* = actual marginal bone level)

Panoramic and periapical radiographic images were used, measuring was performed by calculating proportional length with known length of implant fixture height (H) and measured length (height). Using an image processing software (ImageJ, National Institute of Health), real bone level (X) was calculated by proportional formula [*H*:*X* = *h*:*a*(or *b*)] (Fig. 1). Both mesial and distal aspect of each implants, and also at each side, direct bone–implant contact point (BIC, red point) and distant crestal point (DC, orange point) were the points where drawing a perpendicular line to imaginary extension of implant platform line (black dotted line). Linear measurement of red and orange lines was performed. In this study, bone–implant contact points (BIC) were defined where crestal line (white line) meets implant fixture. Criteria defining distant crestal points are as follows—(1) where horizontally 1 mm distant from the implant platform or the midpoint between implant and adjacent natural tooth; (2) horizontally 1.5 mm distant from the implant platform or the midpoint between two implants; (3) horizontally 1.5 mm distant from the implant which has no adjacent natural teeth nor implants. Where fixture threads were exposed (where marginal bone loss occurred), recorded as positive value, and negative value was recorded when its bone level was higher than the platform of the fixture. The numerical amount of marginal bone change as millimeters were then compared between two groups, focusing on the relative change of bone level from starting point over follow-ups, since the absolute values could be heterogenous due to its variety of implant systems (e.g., trans-mucosal implant versus submerged implant).

## Results

A total of 71 patients, 112 immediately placed implants after tooth extraction initially met inclusion criteria. Demographic data show that 31 patients were male (44 implants) and 40 patients were female (68 implants), in terms of age, 35 patients were elderly those over 65 years (53 implants) and 36 patients were under 65 years (59 implants). 42 patients had at least one underlying systemic disease. 29 patients were not (Table 1).

**Table 1:** Demographic of involved patients and implants

Total 71 patients, 112 implants	Implants (*n*)	Ratio (%)
*Gender*		
Male (31)	44	39
Female (40)	68	61
*Age*		
≥ 65 (35)	53	47
< 65 (36)	59	53
*Underlying*		
Y (42)	66	59
N (29)	46	41

**Fig. 2:**
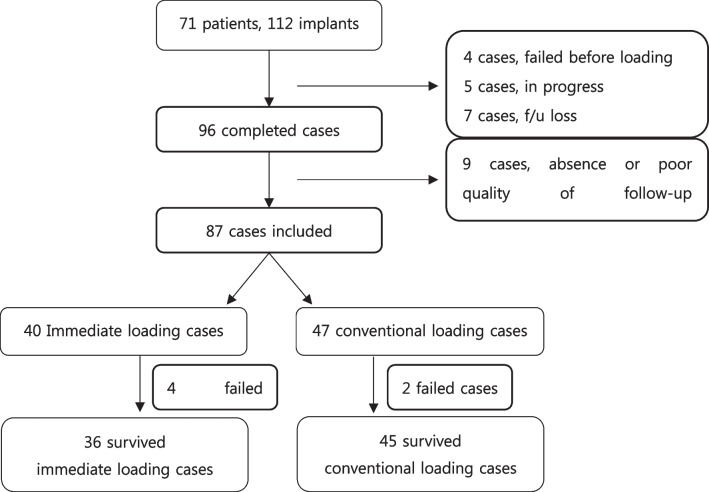
Flowchart showing case selection procedure. Measuring was done with implants which had not failed (81). Failed cases (10) during follow-up period are shown in Table 4 including failure before loading (4)

At implant level, 4 implants failed before prosthetic restoration or loading, 5 implants were not yet finished by definite restorations, 7 implants were lost during the follow-up period before prosthetic restoration. Among 96 completed cases, there were 9 cases which had difficulty to radiographically analyze due to poor quality of either periapical, panoramic view or cone-beam computed tomography, or due to inadequate position of the implants. After the exclusions, 87 cases were finally included (Fig. 2); their information is shown in Tables 2, 3, 4. In the case of bone grafting, only deproteinized bovine bone mineral was used (Bio-Oss®, Geistlich Pharma AG) to fill the gaps between implants and cortical wall of extraction sockets.

**Table 2:** Clinical information of immediate loading (IL) cases

	Implants (*n*)	Ratio (%)
*Immediate loading (IL) (N = 40)*		
Survival		
Survived	36	90
Failed	4	10
Bone grafting		
Y	34	85
N	6	15
Fixture height		
Short (< 8 mm)	0	0
Standard (8 ~ 11.5 mm)	18	45
Long (> 11.5 mm)	22	55
Fixture diameter		
Mini (≤ 4.0 mm)	5	12.5
Standard (4.1 ~ 4.8 mm)	32	80
Large (≥ 5.0 mm)	3	7.5
Implanted site		
Upper anterior	13	32.5
Upper posterior	7	17.5
Lower anterior	7	17.5
Lower posterior	13	32.5

**Table 3:** Clinical information of conventional loading (CL) cases

	Implants (*n*)	Ratio (%)
*Conventional loading (CL)* *(N = 47)*		
Survival		
Survived	45	95.7
Failed	2	4.3
Bone grafting		
Y	37	78.7
N	10	21.3
Fixture height		
Short (< 8 mm)	0	0
Standard (8 ~ 11.5 mm)	37	78.7
Long (> 11.5 mm)	10	21.3
Fixture diameter		
Mini (≤ 4.0 mm)	3	6.4
Standard (4.1 ~ 4.8 mm)	43	91.5
Large (≥ 5.0 mm)	1	2.1
Implanted site		
Upper anterior	2	4.3
Upper posterior	16	34.0
Lower anterior	1	2.1
Lower posterior	28	59.6

**Table 4:** Clinical information of failed cases

Implant distribution	Implants (*n*)	Ratio (%)
*Failed implants (N = 10)*		
Bone grafting		
Y	9	90
N	1	10
Fixture height		
Short (< 8 mm)	0	0
Standard (10 ~ 11.5 mm)	3	30
Long (> 11.5 mm)	7	70
Fixture diameter		
Mini (≤ 4.0 mm)	2	20
Standard (4.1 ~ 4.8 mm)	8	80
Large (≥ 5.0 mm)	0	0
Implanted site		
Upper anterior	5	50
Upper posterior	1	10
Lower anterior	0	0
Lower posterior	4	40
Failed		
After placement, before loading	4	40
After loading		
Immediate loading (IL)		
After provisional restoration	2	20
After final restoration	2	20
Conventional loading (CL)		
After provisional restoration	1	10
After final restoration	1	10

**Fig. 3:**
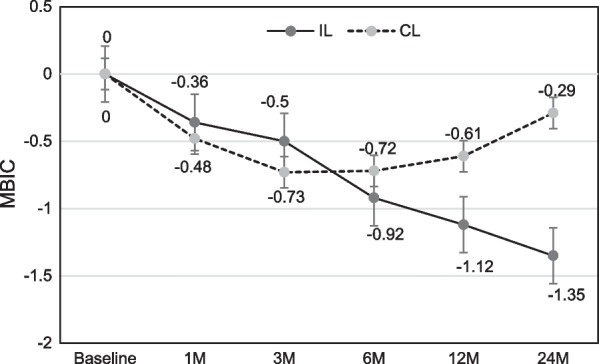
Mean values of marginal bone level over time at mesial bone–implant contact (MBIC) points

**Fig. 4:**
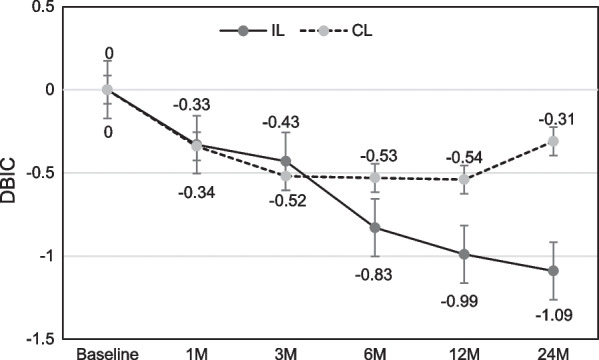
Mean values of marginal bone level over time at distal bone–implant contact (DBIC) points

**Fig. 5:**
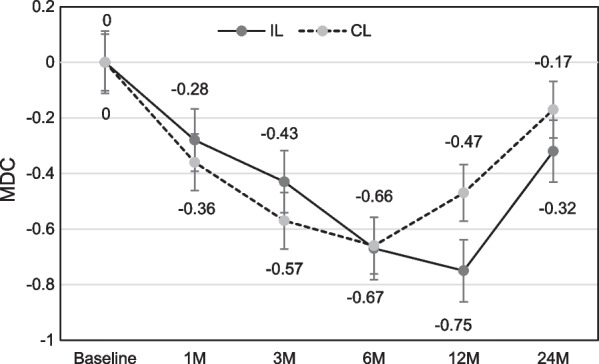
Mean values of marginal bone level over time at mesial distant crestal (MDC) points

**Fig. 6:**
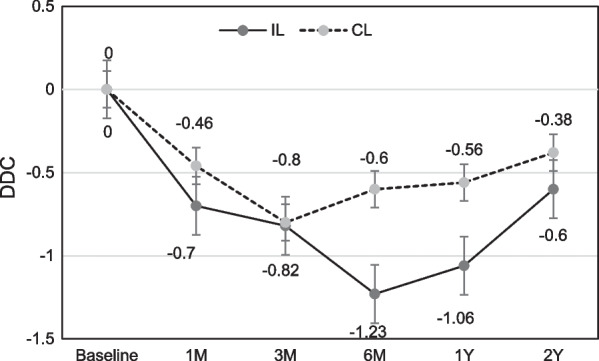
Mean values of marginal bone level over time at distal distant crestal (DDC) points

Marginal bone level (MBL) changes are shown in Figs. 3, 4 as linear graph. Mean values of MBL at direct bone–implant contact surface, both mesial and distal (MBIC, DBIC), showed continuous resorptive tendency in immediate loading group. On the other hand, in conventional loading group, MBL showed significant recovery 3–6 months after the implant placement, although the early period bone loss seems inevitable. At distant crestal points (Figs. 5, 6), also both mesial and distal (MDC, DDC), showed initial bone loss followed by significant recovery 6 months after the implant placement.

## Discussion

Immediate loading implants showed 90% success rate and conventional loading implants showed 95.7%. Both results were close to previous reports of success rates of conventional implants, although the failure rate of immediate implants was slightly higher. This could be due to some risk factors which immediately loaded implants often have. There is always a risk regarding excessive occlusal or lateral force, which can cause micro-movement of implant fixture and deteriorate successful osseointegration [16]. To prevent this, careful and strict case selection must be the priority while planning a rehabilitation treatment and performing delicate and cautious procedure is also necessary [17, 18]. Anterior (incisal) region is where esthetic need is the greatest and its occlusal forces are relatively minimal which mean the concept of immediate provisionalization of immediately placed implants is often needed for saving total treatment time or economic aspect and minimizing the loss of patient’s esthetic. Immediate provisionalization is also can be considered to where bone density could provide sufficient primary stability. On the other hand, patients who have some oral habits which can deteriorate gentle loading of implant prosthesis like clenching or bruxism, must be regarded as high-risk group for immediate provisionalization.

In consideration of the morphology of the extraction socket, the length of implant fixture should be long enough to obtain sufficient initial stability from the floor of the extraction socket. Although there is still no general consensus on the definition of short implant, short implant in this study was defined as implants with a height of less than 8 mm, since there have been constant advances regarding shorter implant hardware and the cut off for short implants has become shorter than previous decade [19, 20]. Among the cases included in this study, no short implant was used, as evidenced by the above reason—sufficient length is necessary for obtaining primary stability in the case of immediately placed implants.

Of the 23 implants at anterior regions of maxilla and mandible, 20 were immediately loaded. Esthetic demand is usually high at anterior maxilla, so often needed for immediate provisionalization. Occlusal forces are relatively low at anterior maxilla, its degree which immediately loaded implants often could withstand, can provides additional clinical options. Since this study has a retrospective nature, it looks natural which is except for only 3 cases, implants immediately placed at anterior maxilla and mandible were immediately loaded because of their relative clinical stability. Twenty of the 64 implants at molar regions were immediately loaded; this relatively low ratio of immediate loading is because of that the high possibility of lateral or excessive occlusal forces at molar regions during mastication. In particular, maxillary molar region usually has low bone density compared to other regions, which makes it hard to obtain sufficient primary stability for immediate loading. Only seven of the 23 implants are immediately loaded.

Of the 112 implants initially included in this study, ten were removed due to failure to achieve and maintain successful osseointegration, four of them removed before loading (during healing periods of implants which were not immediately loaded), another four of them were immediately loaded and removed, two were among conventional loading cases. Criteria defining implant failure included fixture mobility, pain, severe peri-implant radiolucency as described in ICOI 2008 Pisa consensus conference [21]. Among the failed immediate loading cases, three of them were at anterior maxilla, one of them was at anterior mandible. Two of them failed before definite prosthesis delivery. Two failed conventional loading cases failed at the 6 months and 10 months each after the implant placements, both due to the severe peri-implantitis and showed mobility on clinical exam.

Bone grafting is often mandatory when immediately placing an implant after tooth extraction, due to morphological discrepancy between implant fixture and extraction socket. In this study, only the cases which used deproteinized bovine bone mineral as graft material to fill the gaps were present. It is considered as gold standard in alveolar bone graft procedure recent days and the material has adequate radiopacity to be recognized on radiographic image. On the other hand, although in some cases other graft materials such as autogenous bone chips or autogenous fibrin were present, they were inadequate to analyze on radiographic images due to their insufficient radiopacity to be precisely recognized on those images at the day of the surgery, so all the cases which not used deproteinized bovine bone mineral as graft material were excluded due to poor radiographic quality. Although it seems that bone grafting itself does not have a critical role on successful implantation, since nine of the failed ten cases in this study were done with bone grafting simultaneously [22, 23].

With the mean MBL change over time, there was no significant difference between mesial and distal aspect however, its patterns were noticeably different between bone–implant contact and distant crestal aspect. At the direct contact points, there was a loss during initial remodeling phase (3 ~ 6 months), but in the end almost half of the loss was recovered after 24 months past in conventional loading group, although its loss was continued in immediate loading group. This can be thought as successful bone remodeling pattern after implant placement, due to the absence of any obstacles like possibility of early micro-movement during mastication (as in case of immediate or early loading), against normal healing process in conventional loading group. On the other hand, in immediate loading group, continuous bone loss was shown however, the amount of loss is within normal range, as in previous studies have reported [4, 5]. Therefore, it is thought that its continuous loss in immediate loading group is following similar pattern with that of conventional implant protocol, and in conventional loading group it is rather beneficial regarding marginal bone loss compared to the conventional protocol, due to immediate placement after tooth extraction can minimize early bone loss during remodeling phase of extraction socket. Immediate loading group showed 0.92 mm (mean value) more bone loss compared to conventional loading group at bone–implant contact points 24 months after implantation.

At the distant crestal points, both immediate and conventional loading groups showed initial loss and subsequent significant recovery after 24 months past. This pattern seems closer to the pattern which is usually shown at bone remodeling after full thickness mucosal flap (including periosteum) elevation than the pattern in marginal bone change around implant fixture. This might be due to that the effect of different loading protocols was minimal at distant crestal points.

The results can be interpreted that loading time has some effect on peri-implant biological width. Biological width is a natural barrier which can protect submucosal tissue and alveolar bone, and is important for maintaining periodontal health [24]. Although there are some differences between biological width of natural tooth and of dental implant, their functions and importance are similar [25]. In this study, although peri-implant soft tissue was not evaluated, peri-implant marginal bone which consists one of the main components of biological width showed different pattern depending on the loading time.

This study has limitations owing to its retrospective nature, and methodological defect such as absence of standard collimation technique, use of two-dimensional image which cannot represent the whole nature of peri-implant bone or adjacent tissue, not considering differences between various implant systems, and absence of statistical analysis. To analyze the influence of the timing of loading occlusal force to immediately placed implant after tooth extraction, the data from smokers or ex-smokers, as well as patients with uncontrolled systemic disease or metabolic disease were excluded, since those factors could affect the parameters. To investigate the influence of those factors which can be related to the failure of immediately placed implants need to be analyzed in a further controlled study.

## Conclusion

Although this study has limitations owing to its retrospective nature, it is shown that in case of immediate implantation of dental implant after extraction, loading time could affect marginal bone level or biological width of the implant. Immediate loading group showed 0.92 mm (mean value) more bone loss compared to conventional loading group at bone–implant contact points 24 months after immediate implant following tooth extraction. At distant crestal points, there was no noticeable difference in bone change pattern between two groups.

## Data Availability

The datasets used and/or analyzed during the current study are available from the corresponding author on reasonable request.

## Abbreviations

ASA: American Society of Anesthesiologists
MBL: Marginal bone level
IL: Immediate loading
CL: Conventional loading
BIC: Bone–implant contact point (M—mesial, D—distal)
DC: Distal crestal point (M—mesial, D—distal)
ICOI: International Congress of Oral Implantologists
